# Influence of microclimatic ammonia levels on productive performance of different broilers’ breeds estimated with univariate and multivariate approaches

**DOI:** 10.14202/vetworld.2017.880-887

**Published:** 2017-08-08

**Authors:** Essam S. Soliman, Sherif A. Moawed, Rania A. Hassan

**Affiliations:** 1 Department of Animal Hygiene, Zoonosis & Animal Behavior, Faculty of Veterinary Medicine, Suez Canal University, Ismailia 41522, Egypt; 2 Department of Animal Wealth Development, Division of Biostatistics, Faculty of Veterinary Medicine, Suez Canal University, Ismailia 41522, Egypt; 3 Department of Animal Wealth Development, Division of Animal Production, Faculty of Veterinary Medicine, Suez Canal University, Ismailia 41522, Egypt

**Keywords:** ammonia, broiler, discriminant function analysis, growth performance parameters, humidity, temperature

## Abstract

**Background and Aim:**

Birds litter contains unutilized nitrogen in the form of uric acid that is converted into ammonia; a fact that does not only affect poultry performance but also has a negative effect on people’s health around the farm and contributes in the environmental degradation. The influence of microclimatic ammonia emissions on Ross and Hubbard broilers reared in different housing systems at two consecutive seasons (fall and winter) was evaluated using a discriminant function analysis to differentiate between Ross and Hubbard breeds.

**Materials and Methods:**

A total number of 400 air samples were collected and analyzed for ammonia levels during the experimental period. Data were analyzed using univariate and multivariate statistical methods.

**Results:**

Ammonia levels were significantly higher (p< 0.01) in the Ross compared to the Hubbard breed farm, although no significant differences (p>0.05) were found between the two farms in body weight, body weight gain, feed intake, feed conversion ratio, and performance index (PI) of broilers. Body weight; weight gain and PI had increased values (p< 0.01) during fall compared to winter irrespective of broiler breed. Ammonia emissions were positively (although weekly) correlated with the ambient relative humidity (r=0.383; p< 0.01), but not with the ambient temperature (r=−0.045; p>0.05). Test of significance of discriminant function analysis did not show a classification based on the studied traits suggesting that they cannot been used as predictor variables. The percentage of correct classification was 52% and it was improved after deletion of highly correlated traits to 57%.

**Conclusion:**

The study revealed that broiler’s growth was negatively affected by increased microclimatic ammonia concentrations and recommended the analysis of broilers’ growth performance parameters data using multivariate discriminant function analysis.

## Introduction

Housing microclimate is very important for successful farming. Broilers can be reared either on free range extensive system or an intensive system, while for small scale production; a folding unit is the most convenient system. Commercial broiler production requires the construction of a properly designed facility to maintain productivity [1]. Regardless of broiler production system, special attention should be paid to the microclimate: Ventilation, lighting, temperature, humidity, and litter management. Adjustment of temperature values could be made through the ventilation system; however, a great variation in microclimate conditions in broiler’s housing facilities exists with significant effects on body weight, feed intake, and food conversion ratio (FCR) [2].

Poultry farms are natural producers of volatile odorous compounds derived from fermentation, degradation and decomposition of litter and functions of the birds’ organism, such as breathing and digestion, as well as of organic dust which consisted of organic and inorganic particles whose proportion depends on the breed and the production stage of the bird [3]. Production of ammonia resulted in several harmful effects, such as accumulation of nitrogen in aquatic ecosystems strongly related with the loss of biodiversity and soil acidification through oxidation the excessive production of nitrous and nitric acid [4].

The major parameters that influence ammonia concentration in poultry houses are litter conditions and ventilation system. Litter moisture content, pH, and temperature influence the catalyzed degradation of uric acid into ammonia. Bad designed building direction, poor ventilation, incorrect position or fill of drinkers and inadequate litter management are factors contributed in litter with high moisture content in poultry houses. Excessive ammonia levels can negatively affect broiler’s growth and performance traits [5,6] and decrease bird resistance and increase mortality [7].

The study aimed to evaluate the influence of microclimatic ammonia levels on two different breeds of broilers (Ross and Hubbard) reared under different conditions by applying univariate or multivariate approaches and a discriminant function analysis.

## Materials and Methods

### Ethical approval

All applicable international, national, and/or institutional guidelines for the care and use of birds were followed.

### Experimental design

Regular visits on a weekly basis were performed to two broiler farms for two successive fattening cycles. The first farm possessed artificial ventilation; automatic feeding and watering system and consisted of five buildings, each of them with a capacity of 20,000 Hubbard chickens. The second farm had natural ventilation, manual feeding, and watering system and also consisted of five buildings, each of them with a capacity of 5000 Ross chickens. Broilers of both farms were kept on deep litter system (hay). Each rearing period lasted for 38-40 days.

During the visits, average live bird body weight was estimated by randomly weighting a representative number of birds (about 125 birds/each building). At the same time information concerning the weekly feed intake was recorded and the amount consumed by each bird per grams was also estimated. Live body weight and feed intake of the birds were used for the calculation of body weight gain (BWG) [8]; FCR and performance index (PI) [9].

### Measurement and sampling

#### Sampling

A total number of 400 air samples (four samples/week/building/farm/cycle) were collected during the entire experimental period. Air samples were collected using impinger sampler at 50 cm above the ground level to ensure the collection of representative air samples away from the bird’s activity level as recommended. All samples were transported in ice box within 1 h from collection sites to the research laboratory.

#### Microclimatic ammonia levels

The samples were analyzed by potentiometric titration [10] using methyl red indicator and 0.05 M sulfuric acid solution [11].

#### Microclimatic temperature and relative humidity

Temperature and relative humidity of farms were daily recorded during the two fattening cycles (fall and winter) in the two farms (Ross and Hubbard) using an indoor/outdoor-MIN/MAX Thermometer and a Clock Hygro-Thermometer, respectively.

### Statistical analysis

This data were analyzed by different statistical methods using univariate and multivariate approaches. Data were statistically tested for detection of outliers and missing values. Tests of normality for all quantitative variables revealed the normal distribution of the studied biological parameters. Univariate analysis of variance (ANOVA) procedures was conducted to study the effects of microclimatic ammonia levels as varied by fattening cycle (fall and winter); age of bird (from 1st to 5^th^ weeks); temperature and relative humidity levels and broiler breed (Ross and Hubbard) on live body weight, BWG, feed intake, FCR, and PI. The univariate general linear model was fitted as follows:

Y_ijkl_ = µ + B_i_ + F_j_ + W_k_ + BF_ij_ + BW_ik_ + e_ijkl_

Where Y_ijkl_ is the examined variable, µ is the overall mean of the model; B_i_ is the effect of breed; F_j_ is the effect of fattening cycle; W_k_ is the effect of age in weeks; BF_ij_ is the interaction of breed by fattening cycle; BW_ik_ is the interaction of breed by age of bird; and e_ijkl_ is the error. Descriptive statistics of performance traits were presented in addition with their significance tests. Mean separations were done using *post hoc* tests for detection of least significant differences between each pair of means. Bivariate Pearson’s correlation coefficients were estimated for all possible pairs of ammonia, temperature, and humidity.

Multivariate one-way ANOVA (MANOVA) procedure was implemented before discriminant analysis (DA) to study the effect of ammonia in relation to breed on the five examined performance parameters. In MANOVA, breed was the independent variable, while the five performance traits were the dependent variables. The MANOVA procedure was used to test the null hypothesis that the mean vectors of the five traits were equal across the Ross and Hubbard broiler chickens. The null hypothesis was as follows:


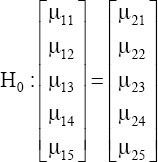


The multivariate model was fitted in analysis to be written as follows:

Y_ijk_ = µ + B_ij_ + e_ijk_

Where, Y_ijk_ is the observation vector related to performance traits j for breed i and k observations; µ is the overall means multivariate vector; B_ij_ is the multivariate vector for the effect of breed i (i=1, 2) on studied variables (performance traits, j=1, 2…, 5); e_ijk_ is the multivariate vector of random errors. The errors vector was assumed to be multinormally distributed, with independently distributed individuals.

Data of Ross and Hubbard broilers were further used in a different statistical approach, namely, the linear DA or canonical DA. This multivariate analysis was aimed to differentiate between Ross and Hubbard broilers based on performance traits. Statistically, DA is a multivariable approach used to predict or classify categorical dependent variable (with two or more level) in the form of linear combination with continuous independent variables [12]. Furthermore, DA was implemented to determine which set of variables best discriminate Ross and Hubbard broilers, with the relative contribution of each variable. DA required exigent assumptions; first, DA assumed equality of variance-covariance matrices (homogeneity) across the grouping variable. This assumption was tested using Box’s M test for equality of covariance matrices. Second, the predictor variables should have multinormal distribution. Third, DA is sensitive to the presence of multicollinearity; hence, independent variables should not be highly correlated. All these assumptions were checked and verified in this study. Discriminant model or function was recorded by Lachenbruch [13] in the form:

D_i_=a+v_1_X_1_+v_2_X_2_+…+v_p_X_p_

Where, D_i_ is the discriminant function score for i breed (i=1, 2), or the canonical variable which was extracted as a linear combination of a set of independent variables (p=5); a is the constant term; v_i_ is the unstandardized canonical discriminant coefficients or weights; X_p_ is the discriminating variables (performance traits). The number of discriminant functions obtained is this study was one because the grouping variable had only two categories (number of functions=number of categories minus one, or the number of independent variables, whenever was smaller). The canonical function was tested for its significance using Wilks’ lambda based on Chi-square statistic, with df equal the number of independent variables. Statistical analysis system version 8.02 [14] and Statistical Package for Social Sciences version 20 [15] were used for conducting all data analyses.

## Results

The influences of ammonia, fattening cycle, and age of bird on performance traits of the two breeds were carried out using a univariate ANOVA. Ammonia levels were significantly higher (p< 0.01) in the Ross compared to the Hubbard farm, although no significant differences (p>0.05) were found between the two farms in body weight; BWG; feed intake; FCR and PI of broilers (Tables-1 and 2). The fattening cycles did not influence ammonia levels (Table 1), but a significant increase (p< 0.01) in body weight, weight gain (Table 1) and PI (Table 2) during the fall was observed, a fact could be attributed to good prevailing weather conditions during the first than the second cycle.

**Table-1:** Effects of breed, fattening cycle, and age of birds (weeks) and their interactions on concentration of ammonia, body weight, and BWG.

Main and interaction effects	Mean±SE		

	Ammonia/ppm	Body weight/g	Weight gain/g
Breed (overall)	p=0.001	p=0.547	p=0.895
Hubbard	7.042^b^±0.048	786.54^a^±15.44	329.56^a^±5.36
Ross	10.752^a^±0.059	785.14^a^±15.40	328.42^a^±5.31
Fattening cycle (overall)	p=0.084	p=0.021	p=0.003
First	8.833^a^±0.139	788.52^a^±16.09	341.97^a^±6.15
Second	8.962^a^±0.145	783.15^b^±14.71	316.00^b^±4.34
Age of bird (overall)	p=0.004	p=0.001	p=0.001
1^st^ week	8.614^b^±0.215	137.14^e^±2.22	102.14^d^±1.44
2^nd^ week	8.908^a^±0.228	370.98^d^±2.43	233.85^c^±1.62
3^rd^ week	9.031^a^±0.217	703.32^c^±1.88	332.33^b^±1.93
4^th^ week	8.963^a^±0.233	1037.79^b^±3.01	334.47^b^±1.76
5^th^ week	8.970^a^±0.231	1679.94^a^±4.56	642.14^a^±4.64
Breed*Fattening cycle	p=0.015	p=0.001	p=0.047
Hubbard*1^st^ cycle	7.069^c^±0.073	794.03^a^±22.92	344.05^a^±8.79
Hubbard*2^nd^cycle	7.016^c^±0.065	779.04^c^±20.69	315.07^b^±6.25
Ross*1^st^ cycle	10.597^b^±0.098	783.01b^c^±22.61	339.89a^b^±8.76
Ross*2^nd^ cycle	10.907^a^±0.061	787.27^b^±20.93	316.94^b^±6.17
Breed*Age of bird	p=0.047	p=0.032	p=0.041
Hubbard*1^st^ week	6.876^c^±0.106	139.29^e^±3.12	104.29^d^±1.95
Hubbard*2^nd^ week	7.144^c^±0.122	369.21^d^±3.48	229.92^c^±2.43
Hubbard*3^rd^ week	7.205^c^±0.093	703.67^c^±2.65	334.45^b^±2.98
Hubbard*4^th^week	6.989^c^±0.116	1037.71^b^±3.97	334.03^b^±2.49
Hubbard*5^th^ week	7.006^c^±0.105	1682.79^a^±6.58	645.08^a^±6.59
Ross*1^st^ week	10.361^b^±0.138	134.99^e^±3.15	99.98^d^±2.21
Ross*2^nd^ week	10.672^ab^±0.191	372.76^d^±3.39	237.76^c^±2.24
Ross*3^rd^ week	10.856^a^±0.104	702.96^c^±2.68	330.20^b^±2.59
Ross*4^th^ week	10.937^a^±0.085	1037.89^b^±4.52	334.92^b^±2.62
Ross*5^th^ week	10.934^a^±0.089	1677.09^a^±6.32	639.20^a^±6.89

Univariate analysis: Means within the same column with different superscripts are considered significant at the 0.05 level of significance (p<0.05). The means separation was conducted by Duncan’s multiple range test. R^2^ for model fitness is 0.87 for ammonia, 0.98 for body weight, 0.95 for BWG, suggesting a good description of the data by the models. SE=Standard error, BWG=Body weight gain

**Table 2:** Effects of breed, fattening cycle, age of birds (weeks) and of their interactions on feed intake, FCR, and PI.

Main and interaction effects	Mean±SE		

	Feed intake/g	FCR/%	PI
Breed (overall)	p=0.747	p=0.826	p=0.768
Hubbard	561.44^a^±8.29	1.760^a^±0.014	0.478^a^±0.011
Ross	564.62^a^±8.62	1.774^a^±0.014	0.472^a^±0.010
Fattening cycle (overall)	p=0.001	p=0.007	p=0.001
First	528.96^b^±8.03	1.679^b^±0.013	0.526^a^±0.013
Second	597.10^a^±8.75	1.855^a^±0.014	0.424^b^±0.008
Age of bird (overall)	p=0.001	p=0.001	p=0.001
1^st^week	156.40^e^±0.767	1.638^c^±0.017	0.093^d^±0.002
2^nd^ week	328.50^d^±2.07	1.463^c^±0.020	0.273^c^±0.003
3^rd^week	603.20^c^±4.54	1.852^b^±0.018	0.399^b^±0.001
4^th^ week	788.70^b^±4.01	2.378^a^±0.013	0.444^b^±0.003
5^th^ week	938.35^a^±3.02	1.504^c^±0.013	1.165^a^±0.011
Breed*Fattening cycle	p=0.001	p=0.117	p=0.414
Hubbard*1^st^ cycle	543.92^c^±11.33	1.722^a^±0.019	0.521^a^±0.018
Hubbard*2^nd^ cycle	578.96^b^±12.29	1.798^a^±0.020	0.435^a^±0.012
Ross*1^st^ cycle	514.00^d^±11.59	1.636^a^±0.020	0.531^a^±0.018
Ross*2^nd^ cycle	615.24^a^±12.66	1.912^a^±0.019	0.413^a^±0.012
Breed*Age of bird	p=0.049	p=0.865	p=0.965
Hubbard*1^st^ week	157.80^e^±1.25	1.592^a^±0.020	0.095^a^±0.003
Hubbard*2^nd^ week	327.80^d^±2.27	1.490^a^±0.029	0.268^a^±0.001
Hubbard*3^rd^week	615.70^c^±6.22	1.895^a^±0.031	0.395^a^±0.001
Hubbard*4^th^ week	778.20^b^±4.76	2.352^a^±0.018	0.449^a^±0.001
Hubbard*5^th^ week	927.70^a^±4.18	1.471^a^±0.015	1.182^a^±0.016
Ross*1^st^ week	155.00^e^±0.95	1.684^a^±0.029	0.091^a^±0.001
Ross*2^nd^week	329.20^d^±3.60	1.435^a^±0.029	0.278^a^±0.001
Ross*3^rd^ week	590.70^c^±6.86	1.808^a^±0.022	0.402^a^±0.001
Ross*4^th^ week	799.20^b^±6.65	2.406^a^±0.021	0.439^a^±0.002
Ross*5^th^ week	949.00^a^±4.50	1.537^a^±0.022	1.149^a^±0.018

Univariate analysis: Means within the same column with different superscripts are considered significant at the 0.05 level of significance (p<0.05). The means separation was conducted by Duncan’s multiple range test. R^2^ for model fitness is 0.87 for ammonia, 0.98 for body weight, 0.95 for BWG, suggesting a good description of the data by the models. SE=Standard error, FCR=Food conversion ratio, BWG=Body weight gain, PI=Performance index

The Ross farm possibly due to the insufficient control of ventilation had significantly higher levels of ammonia (p< 0.01), and these levels were even higher (p< 0.01) in the second (winter) compared to the first (fall) fattening cycle. No significant differences (p>0.05) in ammonia levels between the two seasons were observed in Hubbard farm. A significant increase (p< 0.01) in the levels of ammonia was found for the Ross broilers after the 2^nd^ week of rearing.

Pearson’s correlation coefficient between ammonia, temperature, and humidity are shown in Table 3. A significant positive correlation (r=0.383; p< 0.01) between ammonia levels and ambient relative humidity was found for all birds. However, there was not a significant correlation (r=−0.045; p>0.05) between ammonia levels and ambient temperature. In Hubbard broilers, a significant but weak negative correlation (r=−0.106; p≤0.05) between ammonia levels and ambient temperature was illustrated. No significant correlation (r=−0.081; p>0.05) between ammonia and relative humidity was observed, and this fact could be attributed to the controlled environmental conditions in the farm that reduced ammonia emissions. On the other hand, in Ross broilers, a significant negative correlation (r=−0.268; p< 0.01) between ammonia levels and ambient temperature and a positive correlation (r=0.221; p< 0.01) between ammonia and relative humidity were found. These findings could be a result of the lack of microclimate control as these farms had natural ventilation and not controlled conditions.

**Table-3:** Pearson’s correlation coefficients between ammonia, temperature and humidity for Hubbard, Ross and both breeds.

Breeds	Correlation matrix		

	Ammonia	Temperature	Humidity
Overall data			
Ammonia	1	−0.045^NS^	0.383**
Temperature		1	0.174**
Humidity			1
Hubbard			
Ammonia	1	−0.106*	−0.081^NS^
Temperature		1	0.365**
Humidity			1
Ross			
Ammonia	1	−0.268**	0.221**
Temperature		1	0.010^NS^
Humidity			1

* Correlation is significant at the 0.05 level (two-tailed), where p<0.05.

** Correlation is significant at the 0.01 level (two-tailed), where p<0.01.

NS Correlation is nonsignificant at the 0.05 level (p>0.05)

MANOVA using different indicators, namely, Pillai’s trace, Wilks’ lambda, Hotelling’s trace, and Roy’s largest root revealed no significant (p>0.05) effect of breed on performance traits (Table 4). The value of Wilks’ lambda was high and close to unity (0.999). The MANOVA was used to test the null hypothesis that breed of bird has no significant effect on the all performance traits.

**Table-4:** One-way MANOVA for testing the effect of breed on the performance traits.

Multivariate tests						

Effect	Indicators	Value	F	Hypothesis df	Error df	p value
Intercept	Pillai’s trace	0.995	3617.126	5.000	94.000	0.000
	Wilks’ lambda	0.005	3617.126	5.000	94.000	0.000
	Hotelling’s trace	192.4	3617.126	5.000	94.000	0.000
	Roy’s largest root	192.4	3617.126	5.000	94.000	0.000
Breed	Pillai’s trace	0.001	0.013	5.000	94.000	1.000
	Wilks’ lambda	0.999	0.013	5.000	94.000	1.000
	Hotelling’s trace	0.001	0.013	5.000	94.000	1.000
	Roy’s largest root	0.001	0.013	5.000	94.000	1.000

MANOVA=Multivariate analysis of variance

Although the ordinary MANOVA could describe and test the significant differences between the two broiler breeds, it does not provide information about the best set of predictor variables which may explain the differences between these breeds. DA would be suitable for classification of new classes of birds into their original breeds based on the current data. Results of DA showed the same means and standard deviations of five performance traits within the two breeds. Tests of equality of group mean implemented by DA (Table 5) provided no statistical evidence of significant differences between means of Ross and Hubbard broilers for all the examined performance traits (Wilks’ lambda=1, p>0.05). These results were similar to those obtained by the univariate ANOVA (Tables-1 and 2).

**Table-5:** Tests of equality of group (Ross and Happer breeds) means using canonical discriminant function analysis.

Tests of equality of group means					

Independent variables (predictors)	Wilks’ lambda	F	df 1	df 2	p value
Average body weight	1.000	0.000	1	98	0.990
Average feed intake	1.000	0.003	1	98	0.958
Average BWG	1.000	0.001	1	98	0.976
Average FCR	1.000	0.019	1	98	0.890
Average PI	1.000	0.005	1	98	0.942

FCR=Food conversion ratio, BWG=Body weight gain, PI=Performance index

The pooled within-groups matrices showed that most of the studied discriminatory variables were highly correlated (>0.70). It was found that 60% of correlations (6 of 10 pairs) were high. The presence of high correlations between most of the predictors variables would lead to multicollinearity problem. Further DA would be necessary after removing the highly correlated independent variables. The Box’s M test for equality of covariance matrices revealed no significant (Box M=4.418, F=0.278, and p>0.05), and the log determinants of group covariance matrices were close for the two breeds (18.687-19.02).

One canonical discriminant function was detected in the current data, as the number of discriminant functions to be fitted was equal to the number of groups minus one, or the number of predictor variables, whenever was smaller. The summary statistics of the discriminant function as a linear combination of the five selected variables was presented in Table 6. The value of eigenvalue was lower than one. Canonical correlation which can be defined as the multiple correlation between the discriminant function and the independent variables was 0.026. Testing the significance of the discriminant function was regarded nonsignificant (Wilks’ lambda=0.999, Chi-square statistic=0.066, df=5, and p>0.05).

**Table-6:** Summary of the extracted canonical discriminant function (Eigen value, Wilks’ lambda, and canonical correlation) using all the performance traits.

Eigen value and canonical correlation				

Function	Eigen value	% of variance	Cumulative %	Canonical correlation
1	0.001	100.0	100.0	0.026

**Testing the significance of the discriminant function**				

**Function**	**Wilks’ lambda**	**Chisquare**	**df**	**p value**

1	0.999	0.066	5	1.00

Because DA is negatively affected by multicollinearity as a result of high correlations between independent variables, DA was re-performed several times after the removal of one or more of highly correlated variables (within the lowest F values), and these results were compared with those obtained using all variables of DA. In term of canonical functions, the results showed no significant changes in the summary statistics (Wilks’ lambdas were close to 0.999, p>0.05, with homogeneity of covariance matrices in all analyses), and the only different results were in the classification statistics.

The evaluation of the canonical discriminant function of the present data was implemented by determining the percentage of correct classification of chickens into their original breeds (Table 7). The overall percentage of correct classification was 52% for DA with all the five predictors variables (traits, p=5), with a high percentage of classification error (% of misclassification=48%). On the other hand, the accuracy of classification was improved and increased to 57% for DA with only two traits (p=2), FCR and PI. Although the five traits were phenotypically not important in differentiation between Ross and Hubbard broiler chickens, the removal of the highly correlated variables lead to an improvement in the % of correct classification.

**Table-7:** Classification results with the percentages of correct and incorrect classification.

Discriminant analyses	Correct classification %	Classification error %
DA with all predictors (p=5)	52	48
DA with only FCR and PI (p=2)	57	43

FCR=Food conversion ratio, PI=Performance index, DA=Discriminant analysis

## Discussion

Ammonia is released in broiler farms due to high protein content of diets. Birds cannot store the amino acids consumed beyond their requirements, and their excessive amounts are deaminated and nitrogen is excreted as uric acid (80%), ammonia (10%), and urea (5%) in urine [16]. The large percentage of uric acid is rabidly reduced by uricase and urease enzymes that naturally exist in manure and converted into ammonia. The whole procedure is affected by the prevailed pH and temperature values [17]. The evaporation of ammonia from chicken excreta causes severe environmental problems and leads to the deterioration of manure quality.

Ammonia has usually a characteristic pungent odor that birds can tolerate up to 25 ppm. At high ammonia concentrations; irritation of mucous membranes of the respiratory tract, of the conjunctivae, and of corneas of the eyes occurs. The damage of the respiratory mucous membranes caused by high ammonia concentrations increases bird’s susceptibility to bacterial respiratory infection, especially that of *Escherichia* *coli*. High levels of ammonia also have a negative impact on livability, performance traits as weight gain and FCR, condemnation rate during processing and immune responses of broilers [18].

During the warm months of the year (spring, summer, and early fall) environmental conditions do not affect growth performance parameters since chicken houses are well ventilated. However, during late fall and winter months, if the ventilation system is not properly used, increased ammonia levels can be a problem. These negative effects could be partially alleviated by the use of feed supplements with immune stimulant and antioxidant properties.

Microclimatic levels of ammonia are usually influenced by a number of indoor factors such as temperature, relative humidity, ventilation system, litter condition, and stocking density [19]. Other factors may affect ammonia levels such as heat flow through the ground and environmental air movement. Since the current study was conducted during fall and winter, the low air temperature values induced higher relative humidity levels and as a result elevated levels of ammonia in the naturally ventilated farm compared to the environmentally controlled one. The elevated levels of ammonia resulted in a significant deterioration of the performance traits including BWG, feed conversion, and PI.

Floor of the broiler farms is one of the factors that influence the relative humidity inside the building. In previous studies, deep litter on dirt floors contributed to lower levels of relative humidity (8-10% less), lower levels of ammonia, and less welfare impairment compared to concrete floors [20,21]. In this study; both housing systems had concrete floor that although offers efficient cleaning and disinfection, contributes in a wet litter that is related with increased levels of bacterial fermentation and thus a higher concentration of ammonia. Broiler’s performance is not clearly affected by the floor type as observed by Abreu *et al*. [22], but mortality rates were higher in broiler raised on dirt compared to that raised on concrete floor farms.

Poor maintenance of broiler farms may contribute in a poor management of heating and ventilation that crucially affect the microclimatic temperature, especially in winter seasons and could result in increased levels of ammonia. Good management practices in broiler farms could minimize ammonia levels and its negative effects leading to an improvement of productivity and welfare, reduction of the respiratory diseases incidences and provision of a safe environment for the workers.

The evaluation of the performance traits of broiler chickens by using only univariate analysis could lead to incomplete conclusions due to the fact that biological traits are highly correlated. Hence, the use of multivariate methods becomes necessary for explaining the biological significance of all traits together. Thus, one of the most important limitations of univariate analysis is that it does not explain the covariance between traits. Therefore, in this study, multivariate and canonical discriminant function analysis were further performed to evaluate broiler chicken performance, particularly to discriminate the Ross and Hubbard breeds based on five performance traits. The results of both univariate and multivariate approaches suggested that these five traits are not good discriminatory variables, due to the absence of significant differences between Ross and Hubbard breeds.

Concerning the effects of breed on the five examined performance traits, MANOVA showed the same results as that of univariate analysis [23]. The tests of equality of group means, calculated by canonical DA were also not significant (p>0.05), suggesting that the five variables could not serve for the discrimination between Ross and Hubbard broilers. All the Wilks’ lambda values were close to one, indicating that these performance traits are not sufficient as prediction variables for broiler chickens. The homogeneity assumption of DA was verified in all analyses by Box’s M tests for equality of covariance matrices (p>0.05). The signs of multicollinearity were found among 60% of independent variables and would be expected to affect the results of DA. However, the summaries of canonical discriminant functions were the same in all analyses, even after the removal of correlated traits. The only and positive observed change was in the accuracy of classification. Because DA is a robust test against violation of assumptions [24,25], the summary of discriminant function analysis that is presented in this study (Table 6) include all the five traits. There were sufficient data confirming the inadequacy of the extracted canonical function to explain variations in the grouping variable (breed); the low canonical correlation value, the high Wilks’ lambda value which measure the percentage of total variability and was not explained by the function (99%), and the very low percentage of explained variation (<1%) determined by the discriminant function. Moreover, the nonsignificant (p>0.05) function was a definitive proof for insufficiency of the generated canonical function to discriminate Ross and Hubbard broilers in term of studied performance traits.

In the past few years, many investigations have been conducted with multivariate and discriminant analyses for the estimation of several biological parameters [23,26-28]. However, no research articles are published describing the differences between Ross and Hubbard broilers using a discriminant function analysis. The majority of previous studies were conducted to highlight differences between Ross and other strains, such as Arbor Acres, Cobb 500, and RX. The previous applications of canonical DA in broiler populations have shown high discriminating power between the studied stains [23,29]. Reddish and Lilburn [30] and Rosario *et al*. [23] reported that average live weight is the most appropriate trait to discriminate many chicken populations.

The results of classification confirmed that none of the five examined performance traits was appropriate to distinguish between Ross and Hubbard strains. However, the percentage of correctly classified birds was increased after exclusion of highly correlated variables since the discriminant model only with FCR and PI as independent variables has been proved to increase the accuracy of classification. The percentage of classification error in this study was close to that reported by Shayan *et al*. [12] in a study on cancer patients using DA.

In summary, the results of DA revealed that the five performance traits were poor predictive variables for discrimination between Ross and Hubbard broilers. The results obtained both by univariate and MANOVA procedures were similar. The DA would be more advantageous in describing broiler chicken performance when there are significant differences, to determine the most significant variables in explaining chicken performance. In addition, canonical DA would be used in practice as an alternative multivariate method to understand broiler performance, taking into account the covariance between the studied traits, and determining the relative contribution of each variable in discrimination between breeds.

## Conclusion and Recommendation

The findings of this study revealed that broiler’s growth was negatively affected by variable microclimatic ammonia concentrations under the influences of other contributing factors as the location, housing system and prevailing weather. A good management system is required in broiler farms to adjust the microclimatic conditions with special attention to ammonia. Average live weight, average BWG, average feed intake, average FCR, and average PI appeared to be weak discriminators for differentiation between Ross and Hubbard broilers. Analysis of broiler chicken data using multivariate DA would be informative and permit researchers to explain biological data more extensively compared to the univariate analysis.

## Authors’ Contributions

ESS designed the work scheme and collected data and samples from all farms. RAH assisted in the laboratory work and writing of the manuscript. SAM designed and conducted the statistical analysis. All authors read and approved the final manuscript.
